# Tenecteplase for ischemic stroke at 4.5 to 24 hours without thrombectomy: a cost-utility analysis from the perspective of Chinese healthcare system

**DOI:** 10.3389/fneur.2025.1551332

**Published:** 2025-03-10

**Authors:** Maolin Chen, Ying Yu, Baozhong Yu, Yudan Cao, Yake Lou, Yudong Ma

**Affiliations:** ^1^Emergency Medicine Clinical Research Center, Beijing Chao-Yang Hospital, Capital Medical University, & Beijing Key Laboratory of Cardiopulmonary Cerebral Resuscitation, Clinical Center for Medicine in Acute Infection, Capital Medical University, Beijing, China; ^2^Department of Interventional Neuroradiology, Beijing Tiantan Hospital, Capital Medical University, Beijing, China; ^3^Interventional Center of Valvular Heart Disease, Beijing Anzhen Hospital, Capital Medical University, Beijing, China; ^4^Department of Neurosurgery, Air Force Medical Center, PLA, Beijing, China

**Keywords:** cost-utility analysis, economic evaluation, tenecteplase, stroke, thrombectomy

## Abstract

**Background:**

Tenecteplase improves functional outcomes in acute ischemic stroke (AIS) patients treated 4.5 to 24 h after symptom onset who do not undergo thrombectomy. However, its cost-utility remains unexamined.

**Methods:**

A hybrid model combining a short-term decision tree and a long-term Markov model was developed to simulate the costs and quality-adjusted life years (QALYs) for Chinese patients with AIS at 4.5 to 24 h, who did not undergo thrombectomy. Clinical data were sourced from the TRACE-III trial, while cost data were obtained from the China National Stroke Registry and the Thrombolysis Implementation and Monitor of Acute Ischemic Stroke in China database. The primary outcome was the incremental cost-effectiveness ratio (ICER). Secondary outcomes included total costs, total QALYs and remaining life expectancy, as well as the incremental cost, incremental QALYs, and incremental remaining life expectancy. One-way sensitivity analysis, probabilistic sensitivity analysis (PSA), and scenario analysis were conducted to test the robustness of the results.

**Results:**

For a Chinese patient with AIS treated within 4.5 to 24 h after symptom onset without thrombectomy, adding tenecteplase to standard care resulted in an incremental cost of 2,536 Chinese Yuan (CNY) and an increase of 0.40 QALYs, yielding an ICER of 6,386 CNY per QALY. One-way sensitivity analysis revealed that the most significant factors influencing the ICER were the efficacy and cost of tenecteplase. PSA and scenario analyses confirmed the robustness of these results.

**Conclusion:**

Compared to standard medical treatment alone, administering intravenous tenecteplase between 4.5 and 24 h after onset for Chinese patients with AIS who did not undergo thrombectomy, is highly cost-effective.

## 1 Introduction

Stroke is a leading cause of mortality and disability, particularly in low-and middle-income countries (1). China, the largest middle-income country in the world, bears a significant stroke burden, with ischemic stroke accounting for more than 80% of all strokes (2–4). Timely reperfusion therapy can salvage the ischemic penumbra, improve functional outcome, and reduce mortality and disability in patients with acute ischemic stroke (AIS) (5). Intravenous alteplase or tenecteplase is recommended for patients with AIS within 4.5 h after known onset according to updated guidelines (6, 7). However, only less than 30% of AIS patients arrive at hospital within this critical time frame (8). Mechanical thrombectomy is an effective method to reduce disability and mortality of AIS patients with large vessel occlusion (LVO) within 24 h, especially for those beyond the time window for intravenous thrombolysis and who demonstrate a mismatch between deficit and infarct as assessed by perfusion CT (5, 9). Unfortunately, immediate access to mechanical thrombectomy is limited in many regions, particularly in low-or meddle- income countries with extremely uneven medical resources (10). For patients unable to undergo thrombectomy, the 90-day mortality and disability rates are high as 18.9 and 54.6%, respectively (11). There is still a need to explore effective alternative reperfusion therapy for AIS within the 4.5 to 24-h window.

Tenecteplase, a modified form of human tissue plasminogen activator, has superior fibrin specificity and a longer half-life compared to alteplase, offering an advantage in its administration (6, 12). Recently, the Tenecteplase Reperfusion Therapy in Acute ischemic Cerebrovascular Events-III (TRACE-III) trial firstly investigated the efficacy and safety of tenecteplase in patients with AIS at 4.5 to 24 h after known onset attributed to LVO without endovascular thrombectomy. The results showed that patients treated with tenecteplase administered with 4.5 to 24 h after stroke onset had a better functional outcome (90-days modified Rankin Score [mRS] 0–1: 33.0% vs. 24.2%, relative rate [RR]: 1.37, *p* = 0.03) and comparable mortality (13.3% vs. 13.1%) compared to standard medical treatment (13). However, the incidence of symptomatic intracranial hemorrhage within 36 h was higher (3.0% vs. 0.8%), potentially leading to increased costs. Additionally, tenecteplase use would raise total hospitalization costs. Whether tenecteplase is cost-effective for AIS patients without thrombectomy treated 4.5 to 24 h after onset remains unclear. Our study aims to evaluate the cost-utility of tenecteplase in this population from the perspective of the Chinese healthcare system.

## 2 Methods

### 2.1 Reporting statement and ethical approval

This study is reported in accordance with the CHEERS guidelines (14). Ethical approval was not required for this study, as it utilized publicly available data and did not involve animal research or require informed consent from patients.

### 2.2 Perspective of economic evaluation

This study was conducted from the perspective of the Chinese healthcare system, in the context of the government’s National Volume-Based Procurement program, which aims to lower drug prices and reduce the medication burden on both patients and the national medical insurance system (15). This perspective aligns with the recommendations of the China Guidelines for Pharmacoeconomic Evaluations (16). The analysis focused solely on direct medical costs, excluding indirect medical costs and non-medical costs. Specifically, the cost of tenecteplase was sourced from hospital data and covered by national medical insurance or patients. Additional costs included stroke-related treatment and care, as well as costs associated with adverse events. Indirect costs, such as lost income due to illness, and non-medical costs like transportation, room and board, and nutritional food expenses, were not included. This study aims to provide valuable insights for decision-makers within the healthcare system.

### 2.3 Patients

This study included Chinese patients with AIS who had similar baseline characteristics to those in the TRACE-III trial (13). Specifically, the patients had the following characteristics: age ≥ 18 years (median age 67 years), stroke onset between 4.5 and 24 h, caused by occlusion of the internal carotid artery, middle cerebral artery M1 or M2. Pre-stroke mRS score ≤ 1, baseline National Institutes of Health Stroke Scale (NIHSS) score between 6 and 25, and a target mismatch profile on CTP or MRI + MR Perfusion (ischemic core volume < 70 mL, mismatch ratio ≥ 1.8, and mismatch volume ≥ 15 mL). The patients had not undergone, nor were they planned to undergo, endovascular thrombectomy, and had no contraindications for thrombolysis (17).

### 2.4 Model construction

#### 2.4.1 Approach

**Figure 1:**
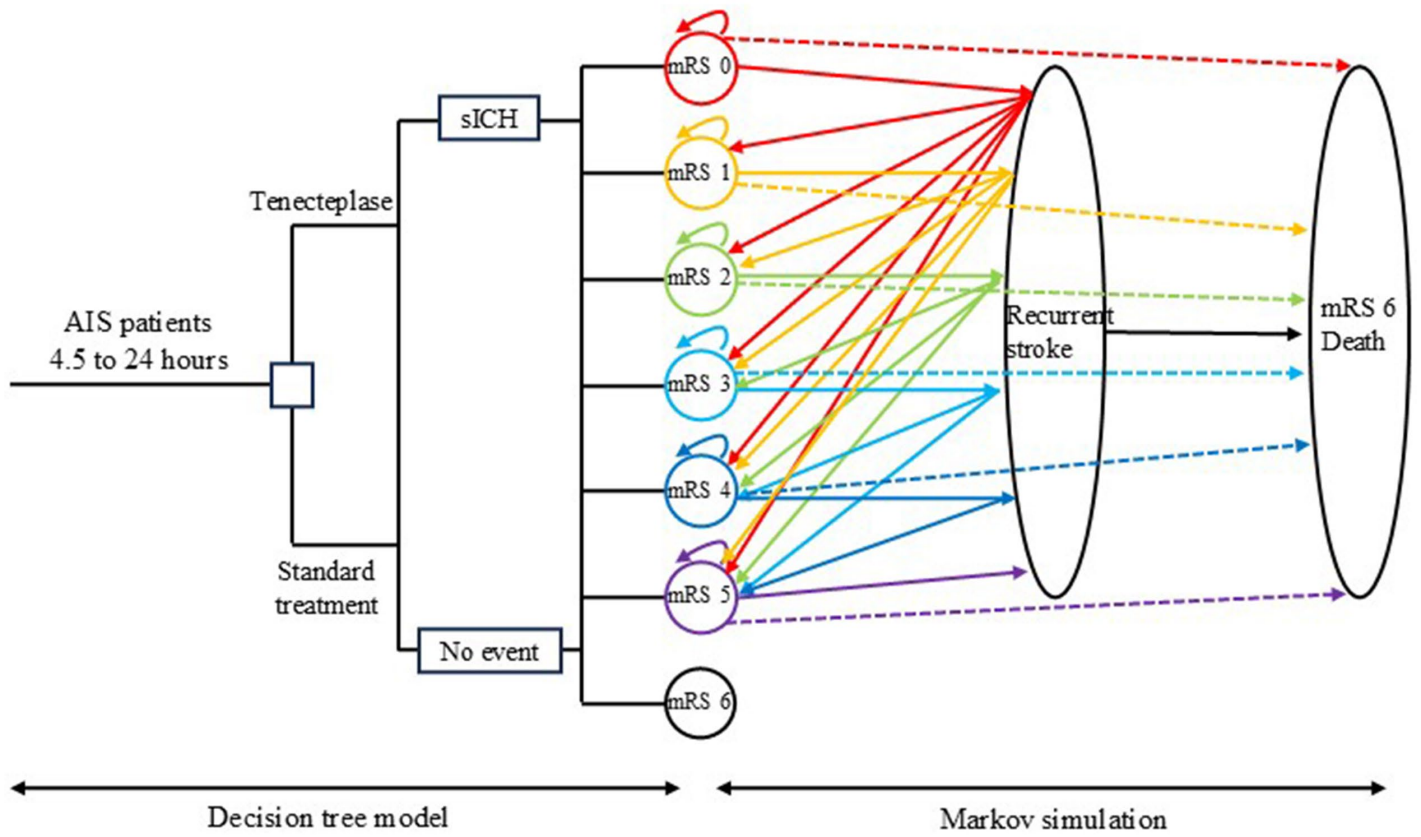
Model diagram. The decision tree model on the left simulates the cost and quality-adjusted life year (QALY) within 3 months after randomization, while the Markov model on the right simulates the long-term cost and QALY. AIS: acute ischemic stroke; sICH, symptomatic intracranial hemorrhage; mRS, modified Rankin scale.

We developed a hybrid model combining a short-term decision tree and a long-term Markov model to simulate the costs and quality-adjusted life years (QALYs) for Chinese patients with AIS occurring 4.5–24 h after onset, who did not undergo thrombectomy. The model compares standard treatment alone with standard treatment plus tenecteplase thrombolysis. Costs and QALYs were derived by summing the results from both the decision tree and the Markov model (Figure 1). To prevent overestimation of costs and QALYs, a half-cycle correction was applied in the long-term Markov simulation.

#### 2.4.2 Model assumptions

Since this is a mathematical model, several assumptions were made to better reflect the natural history of disease progression. Each patient could experience only one clinical event per cycle. If a patient had a recurrent stroke during a Markov cycle, they could not transition to a lower disability level in the subsequent cycle unless they remained stable for at least one full cycle. Patients who survived a recurrent stroke were assumed to be redistributed equally among disability categories of the same or greater severity. Non-stroke mortality was considered for both groups, with rates determined by the mRS classification rather than treatment allocation. Lastly, transition probabilities after discharge were influenced by mRS score and the occurrence of recurrent strokes, regardless of whether tenecteplase was administered during hospitalization.

#### 2.4.3 Model structure

In the decision tree model, eligible patients were randomly assigned to either the standard treatment group or the standard treatment plus tenecteplase thrombolysis group. Patients in the standard treatment group received care according to the 2018 Chinese Guidelines for Diagnosis and Treatment of Acute Ischemic Stroke (18). The tenecteplase group received standard treatment plus tenecteplase thrombolysis. The decision tree model simulated the costs and QALYs at 3 months for AIS patients. Since our study applied the same inclusion and exclusion criteria as the TRACE-III trial, the distribution of mRS scores at 3 months for both groups was based on data from the TRACE-III trial (13).

The Markov model was used to simulate costs and QALYs beyond 3 months. Since patients experiencing a recurrent stroke during a Markov cycle were assumed to be unable to transition to a lower disability level in the subsequent cycle, a cycle length of 3 months was chosen to better reflect the natural history of stroke progression and align with clinical practice. As tenecteplase is administered intravenously as a bolus over 5 to 10 s, its effect is relatively short; however, it can lead to better mRS classification, resulting in lower long-term care costs and improved quality of life after discharge (13). To capture the potential long-term benefits of tenecteplase, a 30-year simulation period was conducted. This duration does not lead to an overestimation of costs and QALYs, as patients who do not survive the full 30 years automatically exit the Markov model and do not contribute additional costs or QALYs. Given the median age of 67 years for patients in our study, the 30-year simulation period exceeds the current life expectancy in China, which is 78 years. The Markov model included seven health states: mRS 0 (no disability symptoms), mRS 1, mRS 2, mRS 3, mRS 4, mRS 5 (severe disability), and mRS 6 (death). The initial mRS distribution in the Markov model was based on the decision tree model, and thus on the TRACE-III trial. In subsequent cycles, patients could experience “no event,” “recurrent stroke” or “death.” Accordingly, in the next Markov cycle, patients could remain in the same mRS state, transition to another mRS state, or move to the absorbing state of “death.” Patients who entered the absorbing state were removed from the model, and no further costs or utility were calculated for them. Patients in mRS states 0 to 5 were simulated in the model until death or up to 30 years. Since the decision tree model simulated the first 3 months of costs and QALYs, the Markov model covered the remaining 119 cycles, for a total simulation period of 30 years.

Symptomatic intracranial hemorrhage (sICH) is a severe complication in AIS patients, and tenecteplase may increase the risk of sICH. Our model incorporated the increased costs and reduced utility associated with sICH.

In both the standard treatment group and the tenecteplase group, the utility was only associated with their mRS state and life year, regardless of whether they received tenecteplase. Similarly, the costs of standard treatment were only related to the mRS state and not to patient grouping.

### 2.5 Intervention

Patients in the standard treatment group received antiplatelet therapy, which included aspirin combined with clopidogrel, aspirin alone or clopidogrel alone, in accordance with the 2018 Chinese Guidelines for Diagnosis and Treatment of Acute Ischemic Stroke (18).

In addition to standard treatment, patients assigned to the tenecteplase group are administered tenecteplase at a dose of 0.25 mg per kilogram of body weight, up to a maximum of 25 mg. Prior to administration, each vial of tenecteplase is dissolved in 3 mL of sterile water for injection and then prepared based on the patient’s weight. The solution is delivered as a single intravenous bolus over 5–10 s (13).

### 2.6 Input parameters

#### 2.6.1 Transition probabilities

In the decision tree model, the 3-month probabilities for mRS scores were derived from the TRACE-III trial (13). In the Markov model, the initial mRS distribution were based on the decision tree model, specifically from the TRACE-III trial (Table 1). The incidence of recurrent stroke was obtained from a Chinese study, reported to be 0.112 annually (19). After a patient experiences a recurrent stroke, within the subsequent 3 months, their mRS state may remain the same, shift to a higher mRS classification, or result in stroke-related death. The probability of stroke-related death following a recurrent stroke is 0.21, based on a study conducted on the Chinese population (20) (Table 1). Like other similar studies, in our study, we assumed that surviving patients after a recurrent stroke would be equally redistributed across the same or more severe disability health states (mRS state) (20–25).

**Table 1:** Key input parameters in the model.

Parameters	Value	Range	Distribution	Source
mRS distribution at month 3 in standard treatment				
mRS 0	0.067	0.036–0.098	Dirichlet	Ref (13)
mRS 1	0.175	0.128–0.221		
mRS 2	0.091	0.056–0.127		
mRS 3	0.234	0.182–0.286		
mRS 4	0.226	0.175–0.278		
mRS 5	0.075	0.043–0.108		
mRS 6	0.131	0.089–0.173		
Risk ratio of mRS distribution in the tenecteplase				
mRS 0	1.46	0.81–2.62	Lognormal	Ref (13)
mRS 1	1.32	0.94–1.87		
mRS 2	1.16	0.69–1.96		
mRS 3	0.74	0.53–1.05		
mRS 4	0.95	0.69–1.32		
mRS 5	0.55	0.27–1.14		
mRS 6	1.01	0.65–1.58		
Probability of sICH in tenecteplase	0.019	0.003–0.035	β	Ref (13)
Probability of sICH in standard treatment	0.008	0–0.019	β	Ref (13)
Recurrent stroke in China	0.112	0.096–0.128	β	Ref (19)
Death after recurrent stroke in China	0.21	0.189–0.232	β	Ref (21)
Death hazard ratios				
mRS 0	1	1–1.2	Lognormal	Ref (25)
mRS 1	1	1–1.2		
mRS 2	1.11	1–1.3		
mRS 3	1.27	1.02–1.52		
mRS 4	1.71	1.37–2.05		
mRS 5	2.37	1.9–2.84		
Background mortality in China				
67–69	0.01266	/		Ref (27)
70–74	0.02159			
75–79	0.03731			
80–84	0.0634			
85-	0.1512			
Utilities				
mRS 0	0.95	0.94–0.96	β	Ref (33, 34)
mRS 1	0.89	0.87–0.96		
mRS 2	0.67	0.54–0.83		
mRS 3	0.44	0.29–0.60		
mRS 4	0.16	0.09–0.23		
mRS 5	0.1	0–0.21		
mRS 6	0	/		
Stroke recurrence in China	0.42	0.11–0.71	β	
Disutility of sICH	0.38	0.30–0.46	β	Ref (21)
Discount rate	0.05	0–0.08	/	Ref (16)
Cost in China				
Tenecteplase (CNY per mg)	230.5	115–459.4	γ	Ref (30)
Acute stroke (mRS 0–1)	12,472	7,204–15,704		Ref (20–22)
Acute stroke (mRS 2–5)	16,490	9,063–21,624		
Acute stroke (death)	14,133	6,640–18,679		
sICH	3,012	654–6,285		
Annual posthospitalization (mRS 0–1)	8,867	2,655–11,311		
Annual posthospitalization (mRS 2–5)	13,492	3,393–16,968		
Recurrent stroke	18,380	13,785–22,976		
IV infusion within 1 h (CNY)	15.6	5–30		Ref (20)

mRS, modified Rankin Scale; sICH, symptomatic intracranial hemorrhage; IV, intravenous; CNY, Chinese Yuan.

In addition to stroke-related death, patients in the Markov model may also experience non-stroke related death (26). Considering that patients with higher mRS grades have a higher risk for non-stroke related death compared to the general population, we adjusted for this using hazard ratios (HR) (25). The background mortality rates for the general population were obtained from the 2022 China Health Statistical Yearbook (27) (Table 1).

All annualized incidence rates were converted to 3-month incidence rates, and these 3-month incidence rates were then converted to 3-month transition probabilities for input in the model. The conversion formulas used were: “*p* = 1 – exp (−r)” and “r = − ln (1 − R)/4,” where p represents the transition probability, r represents the 3-month event rate, and R represents the annual incidence of the event (28) (Table 1).

#### 2.6.2 Cost

All costs in this study are valued in 2023 Chinese Yuan (CNY). For costs incurred before 2023, adjustments were made using the Consumer Price Index (CPI) for healthcare in China. The healthcare CPI for the years 2015 to 2023 was 1.027, 1.038, 1.06, 1.043, 1.024, 1.018, 1.004, 1.006, and 1.011, respectively (29). Future costs were discounted at a rate of 0.05 (range: 0–0.08) as recommended by the China Guidelines for Pharmacoeconomic Evaluations (16).

Since the clinical efficacy of tenecteplase in this study is based on the TRACE-III trial, the cost of tenecteplase was sourced from the same manufacturer as in the TRACE-III trial. The price is based on the government’s National Volume-Based Procurement program initiative by the Chinese government, which is 3,688 CNY per 16 mg (30). This procurement policy covers most public hospitals in China and provides healthcare services to the majority of Chinese patients, making this price representative of the cost for most stroke patients receiving tenecteplase treatment. In this study, the dosage of tenecteplase was 0.25 mg/kg, assuming an average body weight of 75 kg for Chinese stroke patients, with a weight range of 40–100 kg. Additionally, the cost of intravenous injection was included in the analysis (Table 1).

The costs related to stroke treatment were obtained from the China National Stroke Registry (CNSR), which is representative of the overall stroke treatment costs in the Chinese population (20, 31). Stroke treatment costs include acute-phase treatment costs and post-discharge care costs. Acute-phase treatment costs vary according to the mRS classification, with higher mRS scores associated with higher acute-phase treatment costs. Similarly, patients with higher mRS scores incur higher daily care costs post-discharge due to greater disability. Costs for years prior to 2023 were adjusted using the CPI. The cost of recurrent stroke was obtained from a published paper reporting the costs of recurrent stroke in a Chinese healthcare institution (21, 22). Additionally, the extra costs associated with symptomatic intracranial hemorrhage (sICH) were included in the analysis, with the per-case cost of sICH treatment sourced from the Thrombolysis Implementation and Monitor of Acute Ischemic Stroke in China (TIMS-China) database (32) (Table 1).

#### 2.6.3 Utility

Utility reflects the quality of life of patients, and QALY is calculated as life years multiplied by utility. Patients with higher mRS scores have greater disability, resulting in lower quality of life and utility. Even with the same mRS level, utility can vary across different populations. In our study, we used utility values specific to the Chinese stroke population, calculated using the population-based preference weights for each dimension of EQ-5D and applying Chinese preference weights. Negative utility values were applied for sICH and recurrent stroke (33, 34). For future QALYs, the same discount rate used for costs was applied (16) (Table 1).

### 2.7 Outcome

The primary outcome of this study is the incremental cost-effectiveness ratio (ICER) of tenecteplase plus standard treatment compared to standard treatment alone, expressed in CNY per QALY. According to the China Guidelines for Pharmacoeconomic Evaluations, tenecteplase is considered highly cost-effective if the ICER is less than one times the 2023 per capita gross domestic product (GDP) of China, which is 89,358 CNY. If the ICER falls between one to three times the per capita GDP, tenecteplase is considered cost-effective. If the ICER exceeds three times the per capita GDP, it is not considered cost-effective.

Secondary outcomes include the total costs, total QALYs and remaining life expectancy for each treatment group, as well as the incremental cost, incremental QALY, and incremental remaining life expectancy.

### 2.8 Sensitivity analysis

The sensitivity analysis includes both one-way sensitivity analysis and probabilistic sensitivity analysis (PSA). In the one-way sensitivity analysis, a single input parameter is varied within a given range or its 95% confidence interval to observe the fluctuations in the ICER. The results of the one-way sensitivity analysis are presented using a tornado diagram.

In the probabilistic sensitivity analysis (PSA), costs follow a gamma distribution, transition probabilities and utilities follow a beta distribution, initial mRS states follow a Dirichlet distribution, and risk ratios (RR) follow a lognormal distribution. Sampling based on the PSA is performed, and 10,000 Monte Carlo simulations are conducted. The PSA results are presented using an incremental cost-effectiveness scatterplot and a cost-effectiveness acceptability curve.

### 2.9 Scenario analysis

We conducted scenario analyses for several possible situations to further confirm the robustness of our results. These scenarios include using the price of tenecteplase before it was included in the centralized procurement policy, calculating the cost of tenecteplase based on a maximum patient weight of 100 kg, starting age at 60 years and starting age at 75 years.

## 3 Results

### 3.1 Base case analysis

For a Chinese patient with AIS, if only standard treatment is administered, the lifetime cost is 125,229 CNY, with a lifetime QALY of 2.93 (7.30 life years). If the patient receives standard treatment plus tenecteplase, the lifetime cost is 127,765 CNY, with a lifetime QALYs of 3.32 (7.36 life years). The incremental cost is 2,536 CNY, the incremental QALY is 0.40 (0.06 life years), and the ICER is 6,386 CNY per QALY (41,320 CNY per life year), which is below the 2023 per capita GDP of China (Table 2).

**Table 2:** Results of base case and scenario analysis.

	Total cost (CNY)	Total effectiveness (QALY)	Total effectiveness (LY)	Incremental cost (CNY)	Incremental effectiveness (QALY)	Incremental effectiveness (LY)	ICER (CNY per QALY)
Base case							
Standard treatment	125,229	2.93	7.30				
Tenecteplase	127,765	3.32	7.36	2,536	0.40	0.06	6,386
Scenario 1: cost of tenecteplase before government’s National Volume-Based Procurement program							
Standard treatment	125,229	2.93	7.30				
Tenecteplase	132,056	3.32	7.36	6,828	0.40	0.06	17,194
Scenario 2: a maximum patient weight of 100 kg							
Standard treatment	125,229	2.93	7.30				
Tenecteplase	129,205	3.32	7.36	3,977	0.40	0.06	10,014
Scenario 3: starting age = 60 years							
Standard treatment	144,862	3.26	8.56				
Tenecteplase	146,892	3.69	8.60	2030	0.43	0.04	4,730
Scenario 4: starting age = 75 years							
Standard treatment	114,748	2.73	6.63				
Tenecteplase	117,574	3.10	6.70	2,826	0.38	0.07	7,515

CNY, Chinese Yuan; QALY, quality-adjusted life year; LY, life year; ICER, incremental cost-effectiveness ratio.

### 3.2 Sensitivity analysis

**Figure 2:**
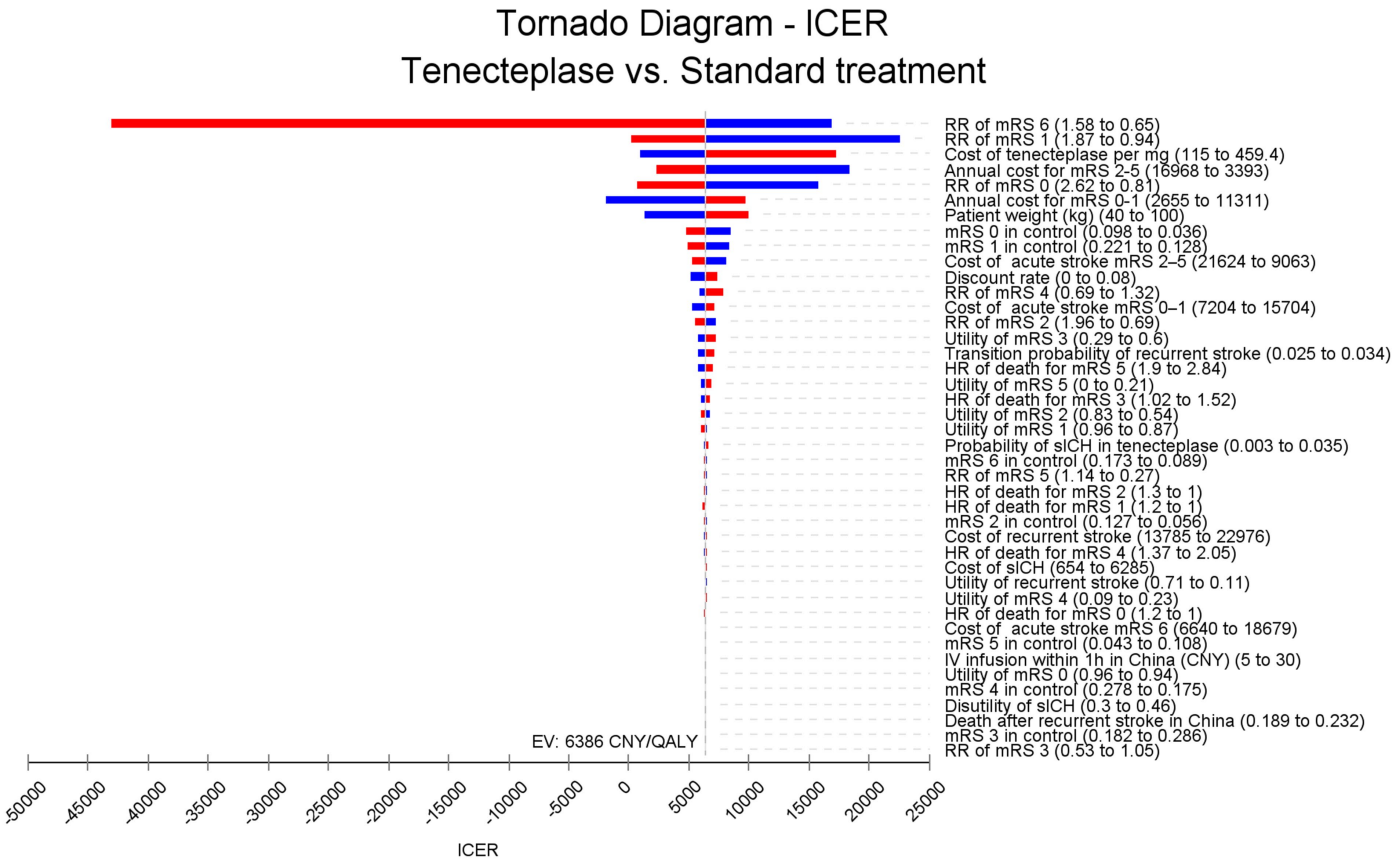
Tornado diagram. The efficacy of tenecteplase, its cost, and the annual cost for patients with mRS 2–5 have the greatest impact on ICER fluctuations. The efficacy of tenecteplase on mortality could drive the ICER below 0, whereas other parameters do not have the potential to lower the ICER below 0. ICER, incremental cost-effectiveness ratio; RR, risk ratio; mRS, modified Rankin Scale; HR, hazards ratio; IV, intravenous; CNY, Chinese Yuan; sICH, symptomatic intracranial hemorrhage. QALY, quality adjusted life year.

Figure 2 shows that in the one-way sensitivity analysis, the efficacy of tenecteplase, its cost, and the annual cost for patients with mRS 2–5 are the most significant factors influencing the ICER. The impact of tenecteplase on mortality in AIS patients (RR of mRS 6) is the most critical factor. When the RR of mRS 6 increases to 1.58, tenecteplase results in lower QALY and higher costs, making it dominated. Although other input parameters influence the ICER, none push it beyond the range of 0–25,000 CNY per QALY.

**Figure 3:**
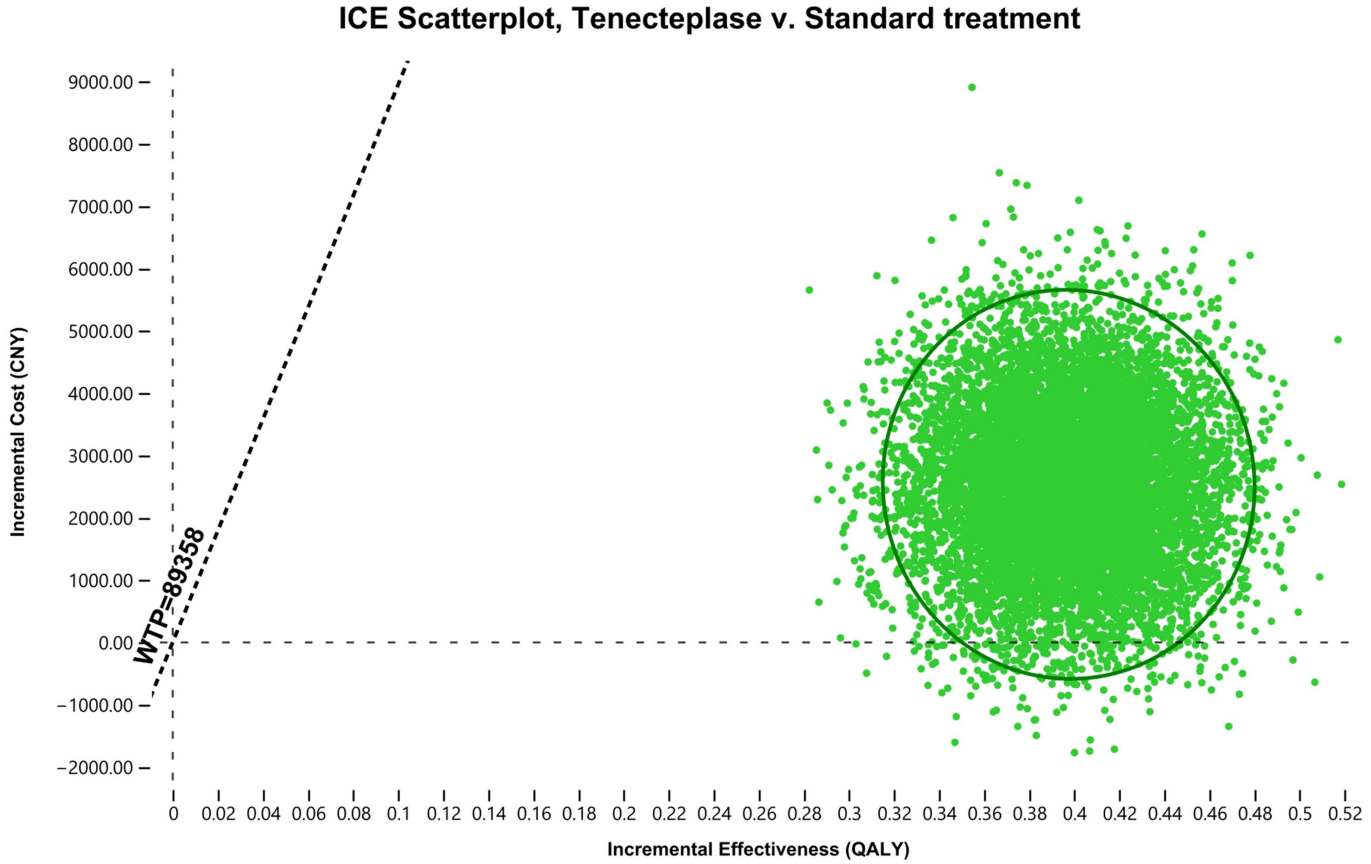
Incremental cost effectiveness scatter plot. All the points in the quadrant are below the WTP threshold line. Approximately 97.7% of the points are in the first quadrant, while 2.3% are in the fourth quadrant. ICER, incremental cost-effectiveness ratio; ICE, incremental cost-effectiveness; WTP, willingness-to-pay; QALY, quality-adjusted life year; CNY, Chinese Yuan.

**Figure 4:**
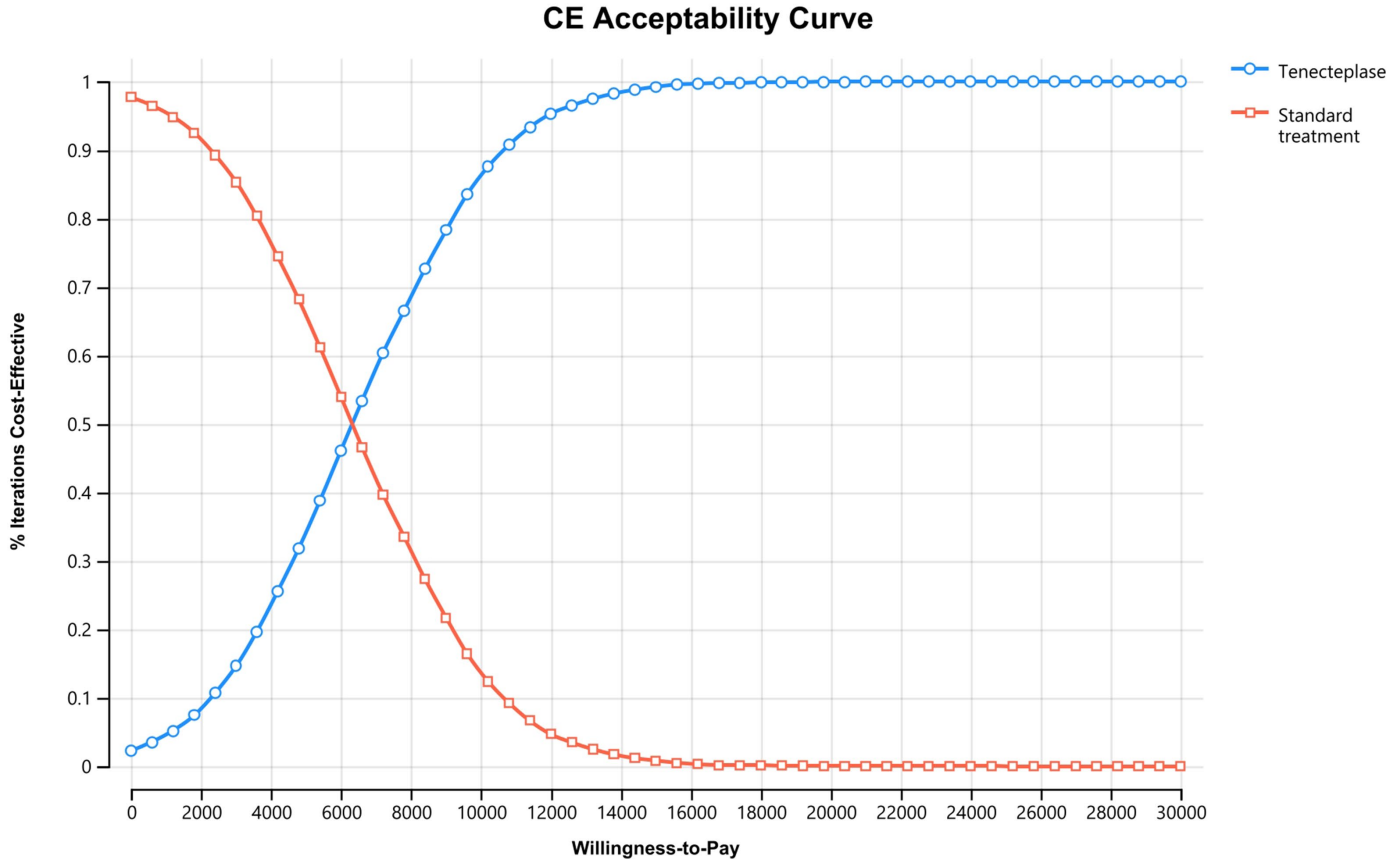
Cost effectiveness acceptability curve. At a WTP threshold of 6,330 CNY per QALY, tenecteplase and standard treatment have similar acceptability. However, tenecteplase becomes more acceptable if the WTP threshold exceeds 6,330 CNY per QALY. CE, cost effectiveness.

PSA results show that in 2.3% of cases, tenecteplase is dominant, meaning it leads to more QALY while incurring lower costs. In 97.7% of cases, tenecteplase is highly cost-effective (Figure 3). The cost-effectiveness acceptability curve indicates that when the WTP threshold is less than 6,330 CNY per QALY, standard treatment is more acceptable. However, when the WTP threshold is 6,330 CNY per QALY or higher, tenecteplase becomes more acceptable (Figure 4).

### 3.3 Scenario analysis

In the scenario where tenecteplase is not included in the government’s National Volume-Based Procurement program, the ICER is 17,194 CNY per QALY, which is well below the WTP threshold. When the maximum dose of tenecteplase is used (25 mg per patient), the ICER is 10,014 CNY per QALY, also well below the WTP threshold. When the starting age in the simulation is set to 60 or 75 years, the ICER remains robust, ranging between 0 and 1 times the per capita GDP.

## 4 Discussion

This study demonstrated that, compared to standard medical treatment, intravenous tenecteplase administered between 4.5 h and 24 h was highly cost-effective for patients with AIS caused by LVO in whom thrombectomy was not available in the Chinese settings. The ICER for intravenous tenecteplase was 6,386 CNY per QALY over a lifetime horizon, which was below the threshold of one time the per capita GDP in China for 2023 (89,358 CNY). What’s more, the results were robust across one-way sensitivity analysis, PSA, and scenario analysis. Our findings support the use of tenecteplase in the late window (4.5 h to 24 h) for AIS patients due to LVO when endovascular thrombectomy is not an option.

The most significant finding of our study was that tenecteplase plus standard medical treatment, as opposed to standard care alone, was highly cost-effective for AIS at 4.5 to 24 h without thrombectomy. According to the tornado diagram, the ICER of tenecteplase was most influenced by the RR of mRS 6 at 90 days and the RR of mRS 1 at 90 days. When the RR of mRS 6 increases to 1.58, tenecteplase results in reduced QALYs and increased costs, making it dominated. However, in a scenario analysis using the highest cost of tenecteplase available on the Chinese market (459.4 CNY per mg), the ICER (17,194 per QALY) remains below the WTP threshold. This suggested that the efficacy of tenecteplase plays a more crucial role than its cost in determining whether tenecteplase is cost-effective for these patients.

The results from the 30-years simulation indicated that tenecteplase provided an incremental QALY of 0.40 with an extra cost of 2,536 CNY. In contrast, a cost-effectiveness analysis of mechanical thrombectomy, based on the data from the DAWN (DWI or CTP Assessment with Clinical Mismatch in the Triage of Wake-Up and Late Presenting Strokes Undergoing Neurointervention with Trevo) trial, reported that mechanical thrombectomy between 6 h and 24 h after symptom onset yielded a lifetime gain of 2.085 QALYs for patients with AIS caused by LVO (9, 25), which was significantly superior to our study. The underlying reasons were as following. Primarily, the efficacy of mechanical thrombectomy in the DAWN trial (mRS 0–2 at 90 days, thrombectomy vs. standard care: 49% vs. 13%) was significantly superior to that of tenecteplase in the TRACE-III trial (mRS 0–2 at 90 days, tenecteplase vs. standard care: 43.6% vs. 33.3%). In addition, the health state utilities of mRS 2–5 used in their study were higher than ours. For patients with AIS due to LVO within 24 h beyond the 4.5 h-time window of intravenous thrombolysis, mechanical thrombectomy is undoubtedly a more effective and cost-effective treatment. However, the effective implementation of mechanical thrombectomy relies on the cooperation of pre-hospital care teams, emergency, radiology, neurology, and neuro-intervention personnel. In low - and middle-income countries with extensive medically underserved regions, delivering thrombectomy may encounter organizational challenges. Tenecteplase is an alternative and cost-effective option to minimize disability for AIS patients unable to undergo thrombectomy in the late window.

Tenecteplase is a genetically modified variant of alteplase, with greater fibrin specificity and longer half-life. Unlike alteplase, which requires an initial bolus injection followed by a 1-h infusion, tenecteplase is administered as a single bolus injection, making it more convenient to use and reduced care cost (35). The EXTEND-IA TNK (Tenecteplase Versus Alteplase Before Thrombectomy for Ischemic Stroke) trial demonstrated that tenecteplase administered before thrombectomy led to better functional outcomes than alteplase in patients with AIS within 4.5 h of onset (36). A subsequent cost-effectiveness analysis base on the EXTEND-IA TNK trial confirmed that tenecteplase, rather than alteplase, can save healthcare system costs (37). Furthermore, the EXTEND-IA TNK Part 2 trial found that 34% of patients in rural areas treated with tenecteplase within 4.5 h after symptom onset achieved substantial reperfusion before thrombectomy, which can reduce medical costs associated with further thrombectomy (38). Two currently underway trials (NCT04454788; NCT05105633) are expected to provide high-quality evidence on the efficacy of tenecteplase as compared to alteplase beyond 4.5 h after stroke onset.

The EXTEND trial, the first study to extend the intravenous thrombolysis time window for alteplase to 9 h, demonstrated that administering alteplase between 4.5 and 9 h after stroke onset resulted in a significantly higher percentage of patients achieving a good functional outcome at 90 days (mRS 0–1) compared to placebo (39). However, there is still a lack of evidence supporting the use of alteplase within the extended time window of 4.5 to 24 h. Tenecteplase has emerged as the first thrombolytic agent being investigated for potential use in this extended time window (4.5–24 h) in patients with ischemic stroke (40). Notably, the TRACE-III trial represents a groundbreaking effort to extend the time window of intravenous thrombolysis to 24 h for patients with LVO who are ineligible for endovascular therapy due to various reasons (13). Two currently underway trials are expected to provide high-quality evidence on the efficacy of tenecteplase compared to alteplase for use beyond 4.5 h after stroke onset, offering hope for advancing treatment strategies in this critical period (41, 42).

Our study has several limitations. First, it is based on a mathematical model rather than actual individual cost data from the TRACE-III trial, which necessitates further investigation for validation. Second, only sICH was included in the analysis, and other complications were not considered. While the occurrence of other complications was comparable between groups, their exclusion could be seen as a limitation of our model. Third, the efficacy and cost data used in the model were derived from China, where there are disparities in healthcare standards between urban and rural areas. This may limit the generalizability of our findings to regions with different healthcare systems. Finally, the efficacy data for tenecteplase were sourced from the TRACE-III trial rather than a meta-analysis of multiple RCTs, so our conclusions should be validated by additional studies.

## 5 Conclusion

From the perspective of the Chinese healthcare system, the addition of intravenous tenecteplase administered for ischemic stroke between 4.5 and 24 h without thrombectomy is highly cost-effective compared to standard medical treatment. However, further real-world studies are needed to validate these findings.

## Data Availability

The raw data supporting the conclusions of this article will be made available by the authors, without undue reservation.
